# Crystalline characteristics of annealed AlN films by pulsed laser treatment for solidly mounted resonator applications

**DOI:** 10.1186/s13065-019-0550-6

**Published:** 2019-03-16

**Authors:** H. K. Lin, Y. J. Huang, W. C. Shih, Y. C. Chen, W. T. Chang

**Affiliations:** 1 Graduate Institute of Materials Engineering, National Pingtung University of Science and Technology, Pingtung, Taiwan; 2 Department of Electrical Engineering, National Sun Yat-Sen University, Kaohsiung, Taiwan; 3 Metal Industries Research & Development Centre, Kaohsiung, Taiwan

**Keywords:** Laser, Acoustic wave device, AlN, Annealing

## Abstract

AlN films were deposited on Si substrates using a reactive RF magnetron sputtering process and then the films were annealed by using different laser powers and wavelengths (355 nm, 532 nm and 1064 nm). For all three laser systems, the (002) peak intensity was obviously improved following laser irradiation. The improvement in the crystalline property was particularly obtained in the AlN film processed at 355 nm. In particular, given the use of the optimal laser power (0.025 W), the (002) peak intensity was 58.7% higher than that of the as-deposited film. The resonant frequency and 3 dB bandwidth of a SMR filter with an unprocessed AlN film were found to be 2850 MHz and 227.81 MHz, respectively. Following laser treatment with a wavelength of 1064 nm and a power of 0.25 W, the resonant frequency changed from 2850 to 2858 MHz. Moreover, 3 dB bandwidth changed from 227.81 to 202.49 MHz and the return loss of the filter reduced from 17.28 to 16.48 dB. Overall, the results thus show that the frequency response of the SMR filter can be adjusted and the return loss reduced by means of laser treatment with an appropriate wavelength.

## Introduction

Acoustic wave devices are widely used in the wireless communication field. Surface acoustic wave filters (SAWFs) [1–3] and film bulk acoustic resonators (FBARs) [4–6] have attracted particular attention as a means of achieving high operating frequencies (exceeding gigahertz) in radio frequency (RF) communications. In realizing such devices, the acoustic wave is confined to resonate as a standing wave using either air gap isolated resonators or solidly mounted resonators (SMRs) [7–9]. SMRs typically consist of a Bragg reflector and a piezoelectric film sandwiched between two electrodes.

The performance of acoustic wave filters is highly dependent on the crystalline quality of the piezoelectric layer. Of the various piezoelectric materials available, single crystal aluminum nitride (AlN) is one of the most commonly used in the optoelectronic, sensor and wireless communication fields due to its wide bandgap (6.2 eV), favorable thermal conductivity (> 100 W/mk) and high dielectric constant (~ 8.5) [10–12]. AlN piezoelectric films can be deposited using various methods, including chemical vapor deposition (CVD) [13], reactive sputtering [14], molecular beam epitaxy (MBE) [15], and pulsed laser deposition (PLD) [16]. However, irrespective of the method used, the quality of the AlN film (and hence the device performance) is strongly dependent on the nitrogen concentration and the processing parameters [15]. Various studies have shown that the crystalline structure of AlN films can be improved through post-deposition plasma, laser, or rapid thermal annealing (RTA) treatment [12, 17, 18]. Lee also reported that an excellent return loss of the solidly mounted resonator-type film bulk acoustic wave resonator devices were observed after the post annealing process [19]. High crystallinity AlN films were obtained by modulating the growth temperature and thermal annealing conditions. High thermal annealing temperature and short annealing time further improve the crystallinity and also preserve the smooth surface [20]. For (10-1-3) and (11-22) AlN layers grown on m-plane sapphire, the crystal quality improved with increasing annealing temperature up to 1700 °C because the density of basal plane stacking faults reduced. These results indicate that the thermal annealing technique offers a new way of fabricating highly efficient semipolar UV LEDs on sapphire substrates [21]. Moreover, laser annealing has been applied in various fields, including active-matrix organic light-emitting diode (AMOLED), metallic glass thin films, complementary metal oxide semiconductor (CMOS), and thin film bulk acoustic wave (TFBAR) [22, 23]. Compared to these traditional annealing techniques, laser annealing has advantages including faster actuation response, rapidly cooling rate and controllable penetration depth into the substrate. It is beneficial for a uniform concentration, lower defect density and local heating area [24]. Cheng [25] reported that laser treatment results in an effective improvement in the c-axis preferred orientation of ZnO films and is thus beneficial in reducing the return loss of ZnO-based longitudinal mode FBARs.

In the present study, the SMR device is consisted by Bragg reflector, AlN thin films and electrode. Firstly, the Bragg reflector is fabricated on Si substrate, and then the bottom electrode is fabricated on Bragg reflector through DC sputter combined with lithography process technology. Secondly, the AlN thin films are deposited on the bottom electrode using reactive RF magnetron sputter. Finally, top electrode is constructed on the AlN thin films by lithography process technology; the SMR device is then completed. The AlN films are then treated by laser irradiation using various laser powers and wavelengths. The effects of the laser processing parameters on the crystalline quality, optical transmittance, resonant frequency and return loss properties of the AlN films are then examined and compared.

## Experiment

**Fig. 1:**
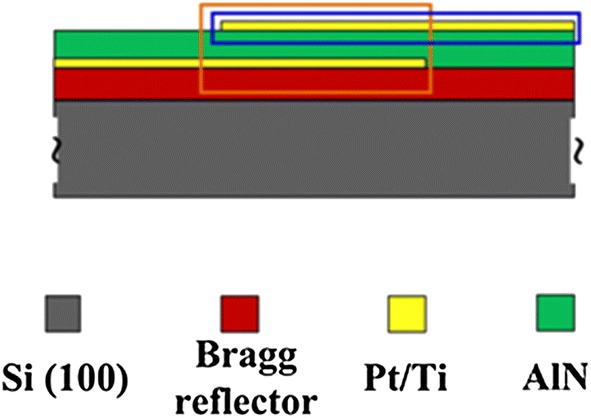
Schematic illustration of SMR filter device

As shown in Fig. 1, the SMR devices fabricated in this study consisted of a Si substrate, a Bragg reflector, an AlN piezoelectric layer, bottom and top electrodes. The AlN film was deposited on the Si substrate using a reactive RF magnetron sputtering system with an Al target. The sputtering process was performed using a N_2_/(N_2_ + Ar) flow of 60%, a base pressure of 5 × 10^−7^ Torr, and a working pressure of 10 mTorr. In addition, the substrate was maintained at a temperature of 300 °C and the sputtering power was set as 230 W.

Following the deposition process, the AlN films were treated using three different laser systems, namely a near infrared laser (SPI G3) with a wavelength of 1064 nm, a Green laser (Coherent MATRIX 532-7-30) with a wavelength of 532 nm, and a UV laser (Coherent AVIA 355–7000) with a wavelength of 355 nm. In laser patterning processes, the diameter of the laser spot, D_0_, can be calculated as

1 $$D_{0} = 1.22 \times \left( {\frac{\lambda \times F}{{n \times W_{d} }}} \right) \times M^{2} ,$$

where λ is the laser wavelength, *F* is the focal length, *n* is the refractive index of the working material, *W*_*d*_ is the diameter of the incident laser beam, and *M*^2^ is the laser quality factor. For the NIR laser, *M*^2^ was equal to 1.3, *F* was 163 mm, and *W*_*d*_ was 7.2 mm. Meanwhile, for the Green laser, *M*^2^, *F* and *W*_*d*_ were equal to 1.3, 330 mm and 5.8 mm, respectively. Finally, for the UV laser, *M*^2^ was equal to 1.3, *F* was 100 mm, and *W*_*d*_ was 3.5 mm. The spot diameters for the three laser systems were thus equal to 40, 50 and 17 μm, respectively. For each system, the laser power was set in the range of 0.01–1.5 W and the scanning speed was fixed at 5 mm/s.

The relative enhancement in the XRD peak intensity, I_R_, can be quantified as

2 $${\text{I}}_{\text{R}} = \left( {{\text{I}} - {\text{I}}_{\text{o}} } \right)/{\text{I}}_{\text{o}} ,$$

where I_R_ is the relative enhancement in the XRD peak intensity, I is the intensity following laser treatment, I_o_ is the initial intensity.

The surface morphologies of the various samples were observed using a field emission scanning electron microscope (FESEM, JSM-7600F). Moreover, the microstructures of the samples were examined using an X-ray Diffractometer (Bruker D8) with Cu-Ka radiation. The optical transmittance was measured using a UV–VIS-IR spectrophotometer (Lambda 35). Finally, the frequency response of the SMR filters was measured using a network analyzer (E5071C) and a CASCADE high-frequency probe (RHM-06/V + GSG 150).

## Results and discussions

**Fig. 2:**
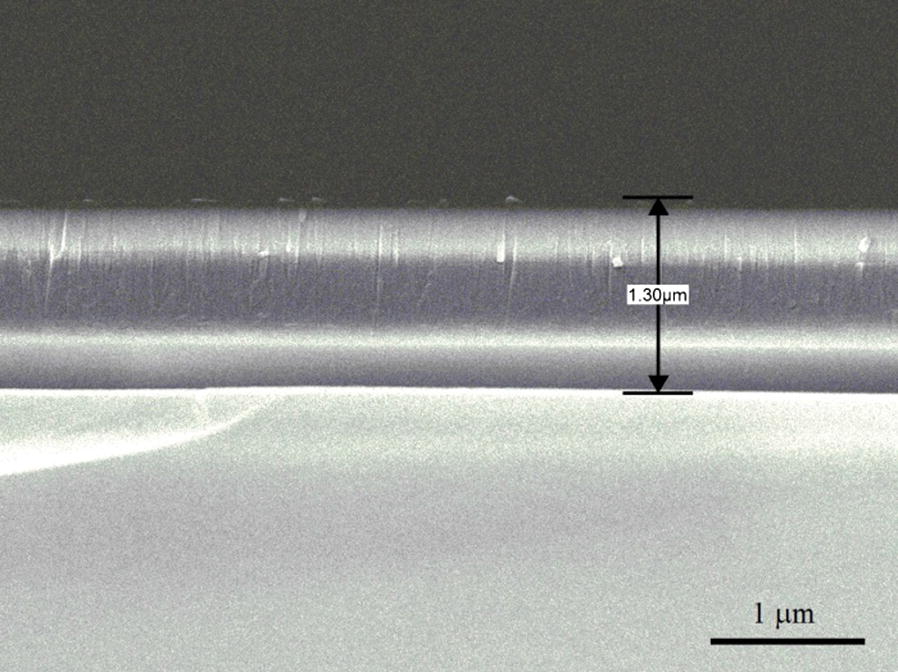
SEM cross-sectional image of as-deposited AlN film

**Fig. 3:**
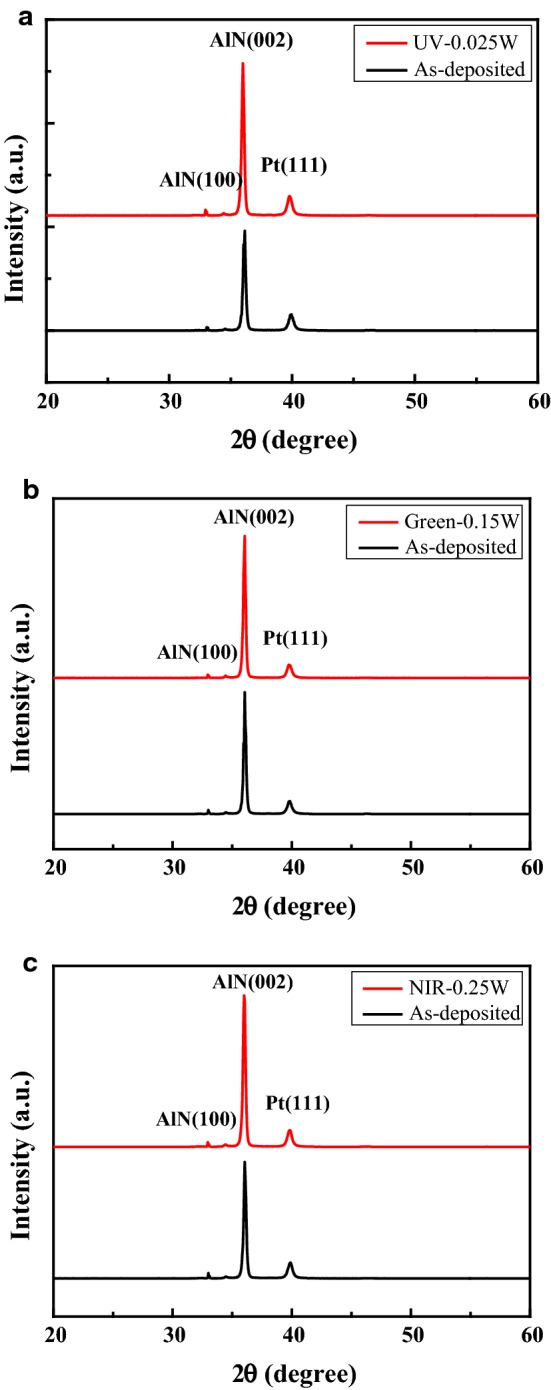
XRD profiles of as-deposited AlN film and AlN film annealed using three laser systems: **a** UV, **b** green and **c** NIR

Figure 2 presents a cross-sectional SEM image of the as-deposited AlN film. As shown, the film has a thickness of approximately 1.3 μm. Figure 3 shows the XRD spectra of the as-deposited AlN film and the film annealed by these laser systems. It is seen that the preferred (002) orientation of the AlN film is obtained in both samples. However, it is evident that the annealing process results in a significant increase in the (002) peak intensity. The UV annealing process increases the (002) peak intensity by 58.7% compared to that of the as-deposited AlN sample. The relative improvement for the Green and NIR system is seen to be 16.8% and 36.8%, respectively.

**Fig. 4:**
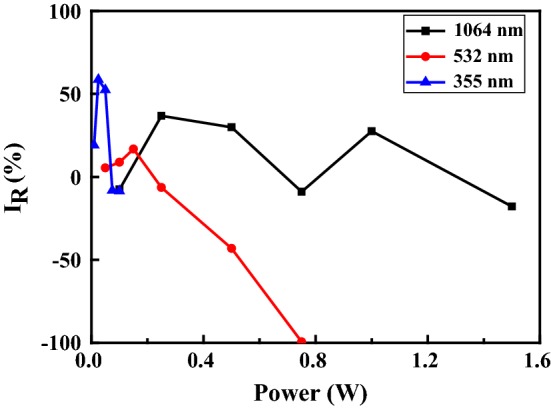
Relative improvement in (002) peak intensity as function of laser power for laser systems with different wavelengths

**Fig. 5:**
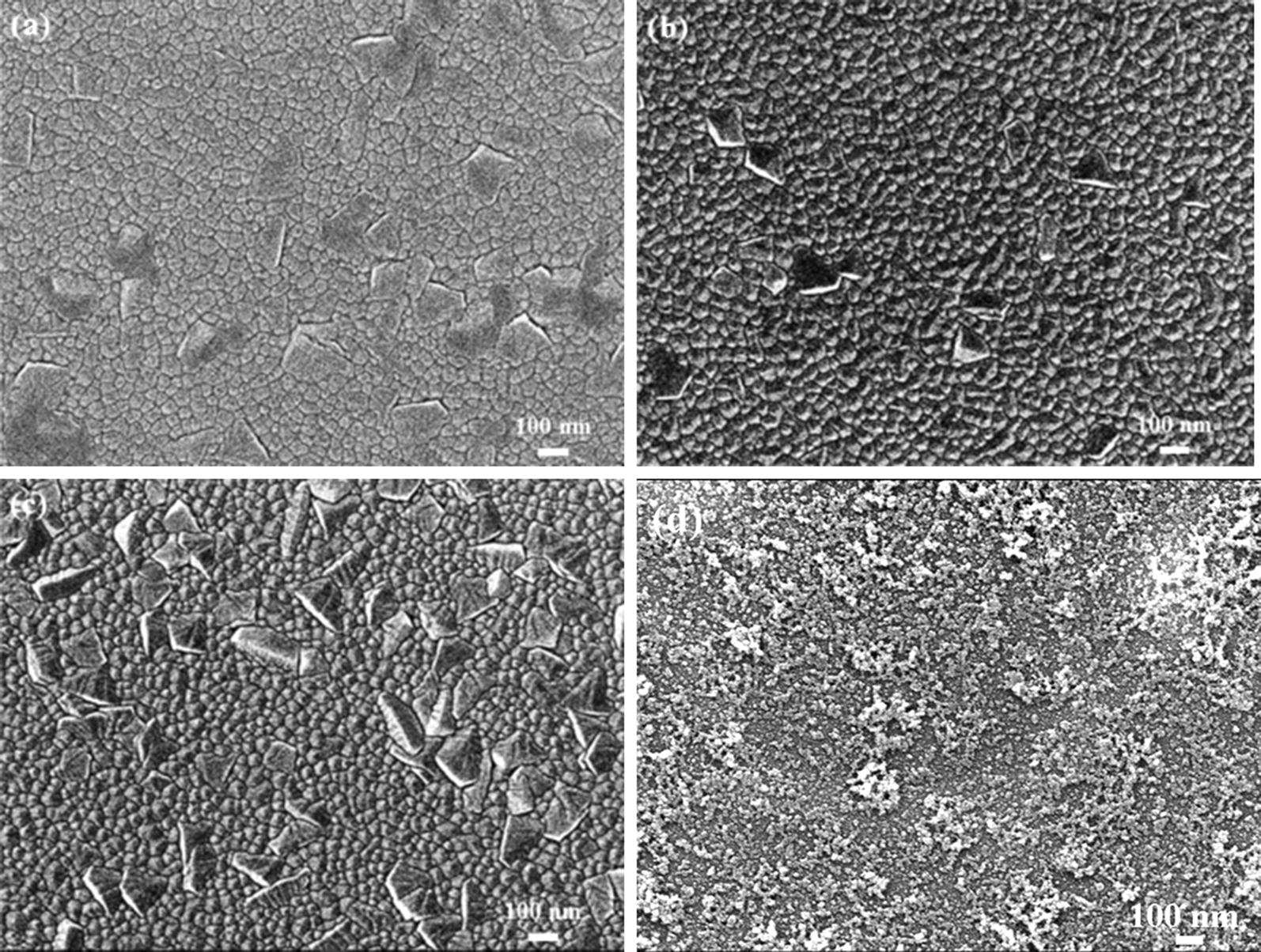
SEM images of as-deposited and UV-annealed AlN films: **a** as-deposited, **b** 0.025 W, **c** 0.05 W and **d** 0.1 W

Figure 4 shows the relative enhancement in the (002) peak intensity as a function of the laser power for all three laser systems. It is apparent that the effectiveness of the different laser systems in improving the crystal quality of the AlN film is extremely sensitive to the processing power. From inspection, the optimal powers of the UV (355 nm), Green (532 nm) and NIR (1064 nm) laser systems are equal to 0.025 W, 0.15 W and 0.25 W, respectively. Given these values of the laser power, the relative improvement in the (002) peak intensity is seen to be 58.7%, 16.8% and 36.8%, respectively. Figure 5 presents SEM images of the as-deposited AlN film and the AlN films processed by the UV laser with various powers. For laser powers lower than 0.05 W, the AlN film has a dense microstructure with a strong c-axis orientation. In other words, a recrystallization of the as-deposited film occurs following the laser treatment process. However, for powers greater than 0.1 W, the AlN films have a cracked and rough surface due to the ablation effect caused by the higher thermal input energy. As a result, the (002) peak intensity of the AlN film is significantly reduced, as shown in Fig. 4.

**Fig. 6:**
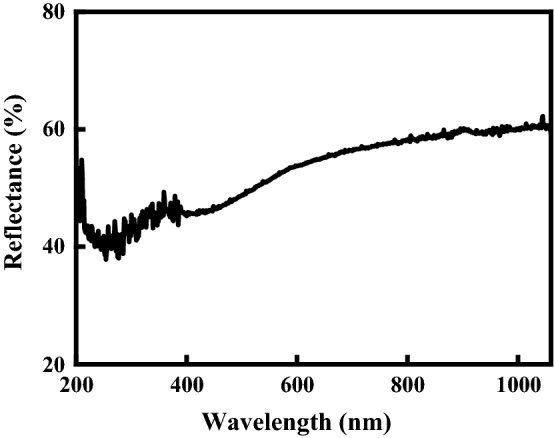
Reflectance spectrum of the as-deposited AlN film

**Fig. 7:**
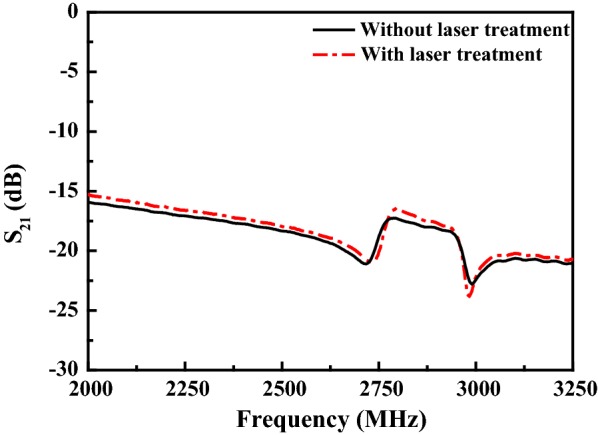
Resonant frequency of SMR filters with as-deposited and annealed AlN films

Figure 6 shows the reflectance spectrum of the as-deposited AlN film. From inspection, the reflectance values of the AlN films processed using the NIR, Green and UV laser systems are 61, 51 and 46%, respectively. In other words, the absorption of the AlN film increases with a decreasing wavelength. It is noted that this finding is consistent with the results presented in Fig. 4, which show that the optimal power (0.025 W) for the UV system (wavelength 355 nm) is lower than that (0.25 W) of the NIR laser system (wavelength 1064 nm). Figure 7 shows the center frequency response of two SMR filters containing an as-deposited AlN film and an AlN film annealed using the NIR laser system with a power of 0.25 W, respectively. The SMR filter containing an unprocessed AlN film has a resonant frequency of 2850 MHz. However, following laser irradiation, the resonant frequency increased to 2858 MHz, while the frequency of 3 dB bandwidth changes from 227.81 to 202.49 MHz. In addition, the return loss of the SMR filter decreases from 16.48 dB in the untreated condition to 17.28 dB in the annealed condition as a result of the improved crystalline quality of the AlN film. Overall, the results show that the resonant frequency and return loss of the SMR filter can both be adjusted through the use of an appropriate laser treatment process.

## Conclusions

AlN films have been deposited on Si substrates using a reactive sputtering process. The AlN films have been laser annealed with various laser powers and wavelengths. The XRD results have shown that the laser annealing process yields an effective improvement in the (002) peak intensity of the AlN film. For irradiation wavelengths of 355, 532 and 1064 nm, the optimal laser power has been found to be 0.025, 0.15 and 0.25 W, respectively. The corresponding enhancement in the (002) peak intensity is equal to 58.7%, 16.8% and 36.8%, respectively. For a SMR filter with an as-deposited AlN film, the resonant frequency is equal to 2850 MHz and the 3 dB bandwidth is 227.81. Following laser treatment with a wavelength of 1064 nm and a power of 0.25 W, the resonant frequency increases to 2858 MHz. In addition, the 3 dB bandwidth reduces to 202.49 MHz and the return loss reduces from 17.28 to 16.48 dB. In other words, the resonant frequency of the SMR filter can be tuned and the return loss reduced through the use of a laser irradiation process with appropriate wavelength and power settings.
